# Verdiff-Net: A Conditional Diffusion Framework for Spinal Medical Image Segmentation

**DOI:** 10.3390/bioengineering11101031

**Published:** 2024-10-15

**Authors:** Zhiqing Zhang, Tianyong Liu, Guojia Fan, Yao Pu, Bin Li, Xingyu Chen, Qianjin Feng, Shoujun Zhou

**Affiliations:** 1 Shenzhen Institute of Advanced Technology, Chinese Academy of Sciences, Shenzhen 518055, China; zq.zhang3@siat.ac.cn (Z.Z.);; 2 University of Chinese Academy of Sciences, Beijing 100049, China; 3 Institute of Unconventional Oil & Gas Research, Northeast Petroleum University, Daqing 163318, China; 4 College of Information Science and Engineering, Northeastern University, Shenyang 110819, China; 5 Department of Health Technology and Informatics, The Hong Kong Polytechnic University, Kowloon, Hong Kong SAR, China; 6 School of Biomedical Engineering, Southern Medical University, Guangzhou 510515, China

**Keywords:** spinal segmentation, diffusion model, multi-modality

## Abstract

Spinal medical image segmentation is critical for diagnosing and treating spinal disorders. However, ambiguity in anatomical boundaries and interfering factors in medical images often cause segmentation errors. Current deep learning models cannot fully capture the intrinsic data properties, leading to unstable feature spaces. To tackle the above problems, we propose Verdiff-Net, a novel diffusion-based segmentation framework designed to improve segmentation accuracy and stability by learning the underlying data distribution. Verdiff-Net integrates a multi-scale fusion module (MSFM) for fine feature extraction and a noise semantic adapter (NSA) to refine segmentation masks. Validated across four multi-modality spinal datasets, Verdiff-Net achieves a high Dice coefficient of 93%, demonstrating its potential for clinical applications in precision spinal surgery.

## 1. Introduction

**Figure 1:**
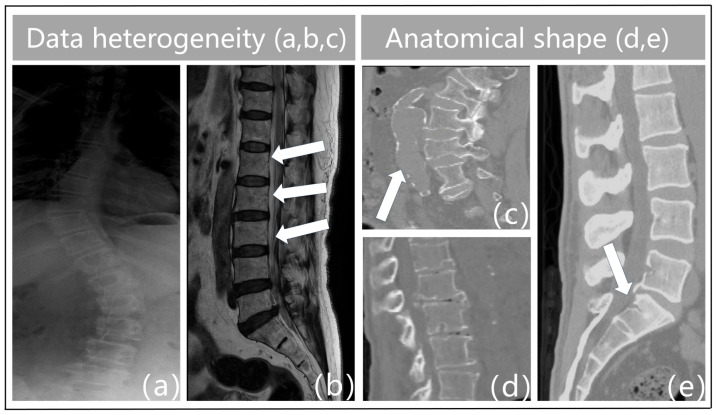
Challenges in resolving multi-modality vertebral images include: (**a**) boundary-blurring from X-ray imaging methods. (**b**) interclass similarity when vertebrae are present in MR. (**c**) The misdiagnosis in CT imaging of vertebrae and adjacent tissues with similar CT values. (**d**,**e**) weak vertebral contouring from the encroachment of vertebral degeneration/disease.

Spinal surgical conditions are prevalent and frequently result in high rates of disability, significantly diminishing patients’ quality of life. They have gradually emerged as major health concerns, profoundly impacting individuals’ daily lives [1,2,3]. An essential first step toward a thorough diagnosis and treatment of spinal illnesses is the automated extraction of the vertebral form from various vertebrae medical pictures. Specifically, the evaluation, diagnosis, surgery planning, and image-guided interventional processes of numerous vertebrae illnesses depend on the precise segmentation of vertebrae and intervertebral disks (IVDs) in vertebrae images. Extensive research efforts have been made by several researchers to enhance the segmentation performance of models for vertebrae images. Specifically, the methods in this field are mainly divided into two categories, such as single-modality and multi-modality. The single-modality approaches [4,5] are common and have drawbacks in clinical practice, and frequently necessitate an extended development cycle. A multi-modal approach [6] can leverage data from multiple imaging modalities at the same time, each of which offers distinct anatomical and pathologic details that contribute to a more thorough reveal of the spinal system and its diseases. Furthermore, by minimizing the dependence on a single imaging technology, the multi-modality approach lowers the possibility of misdiagnosis and enhances diagnostic accuracy and reliability through thorough analysis. However, investigations on multi-modality vertebrae image segmentation techniques in clinical settings are still very scarce. Meanwhile, researchers must overcome the following two primary obstacles in order to produce accurate and dependable multi-modality vertebrae segmentation results because of the variety of imaging modalities used on spinal medical pictures as well as the distinctiveness of their anatomical structures:

**(1)**  **Data heterogeneity.** The term “heterogeneity of data” describes the variety and diversity of the data, or the variations in the data across various dimensions. These variations could be caused by a variety of things, including the characteristics of the chiropractic data itself, the acquisition technique, the equipment used, the duration of the acquisition, and more. For instance, feature extraction varies throughout different types of imaging data (e.g., MRI, X-ray, CT, etc.), and variations in acquisition equipment can result in issues with data quality such as noise and distortion in vertebrae imaging. Figure 1a illustrates the blurred outlines caused by the physical properties of X-ray imaging. Second, interclass similarity is seen in spinal MR images [5]. This means that neighboring vertebrae (intervertebral disks) in the same subject (Figure 1b) exhibit a high degree of morphological resemblance, making it more challenging to distinguish between individual vertebrae. The surrounding tissues in Figure 1c have similar physics and tissue densities to the vertebrae in CT imaging, which leads to identical CT values that confuse the background features and result in erroneous detections. Analogously, variations in the duration of acquisition may cause data drift and variability. As a result, data heterogeneity must be taken into account and managed throughout data processing and analysis since it is one of the key elements influencing the outcomes of data analysis and mining.
**(2)**  **Anatomical shape.** Vertebral pictures show features such as blurriness, uneven grayscale distribution, high noise levels, and low contrast because of the state of spinal medical imaging today. The vertebrae that make up the spinal structure also have a similar form but different types. Spinal illnesses like vertebral strain alter the anatomical form of the vertebral bodies, as shown in Figure 1d. Furthermore, the vertebrae are spatially displaced as a result of trauma, bad posture, muscular imbalance, and congenital deformities [7]. This causes aberrant modifications or misalignment of their locations in space, which severely distorts the morphology of the vertebral bodies. Particularly, in Figure 1e, the individual had fractures or breaks in the sacral vertebrae as a consequence of external pressures. This may lead to further deformation, which makes the connections between the lumbar and sacral vertebrae extremely tight. As such, defining the borders between these joint vertebrae based on pixel intensity is difficult. This frequently results in semantic segmentation of these linked vertebrae as a single object during vertebral segmentation, which causes misidentification as a single vertebra. Given the increased unpredictability in the contour forms and placements of the vertebral bodies, these features surely make vertebral segmentation tasks more challenging.

Most of the existing deep learning (DL)-based image segmentation methods perform prediction and discrimination by directly learning the probability of classifying image pixels, and these methods fall into two main categories: convolutional neural network (CNN)-based methods [8,9] and visual Transformer (ViT)-based methods [10,11,12]. These methods typically use the cross-entropy or Dice loss function to train a model that learns a mapping function from an input medical image to a segmentation mask. Although these methods have been effective in specific applications, they mainly focus on learning decision boundaries in the pixel feature space [13], ignoring the distributional properties of the underlying data [14] and failing to adequately capture the intrinsic class characteristics of the data. Additionally, the learned feature space of existing methods exhibits a rapid decline in performance in regions far from the decision boundary [15], posing challenges for handling fuzzy boundaries and fine objects. This instability limits the model’s ability to recognize subtle variations in images, which is particularly significant in medical image segmentation where these variations can be crucial for diagnosis and treatment. Therefore, despite the progress made by CNN- and ViT-based methods in the field of image segmentation, there is still room for improvement in terms of deeply understanding data distribution and enhancing the stability of the feature space. Generative models [16,17,18,19,20] discover intricate probabilistic correlations between images and segmentation masks in the field of medical image segmentation. Instead of producing a fixed segmentation result, these algorithms generate a probabilistic distribution that directly models the underlying data distribution and predicts the segmentation mask conditional distribution. On the other hand, this method is more likely to capture image ambiguity [17,21] and produce segmentation results that are smoother and more reliable.

Diffusion probabilistic models are renowned as powerful generative models, possessing excellent capabilities for modeling (un)conditional data distributions. This brings significant advantages to the field of medical image segmentation. In practice, researchers have adopted two strategies: on the one hand, diffusion models are directly applied to treat image segmentation as a generative task, leveraging DDPM’s ability to capture fine details in data distribution [20,22,23]. On the other hand, some studies aim to integrate the precision of discriminative methods with the creativity of generative methods [17,24,25], striving for superior performance in segmentation tasks. However, existing methods still have shortcomings in integrating the features of input data and segmentation masks. They often adopt a rather coarse concatenation approach when combining these two types of features for joint probabilistic modeling, leading to the loss of critical information. Moreover, although these methods are effective in general scenarios, they still require further customization and optimization in specific application contexts to meet unique demands and address corresponding challenges.

**Figure 2:**
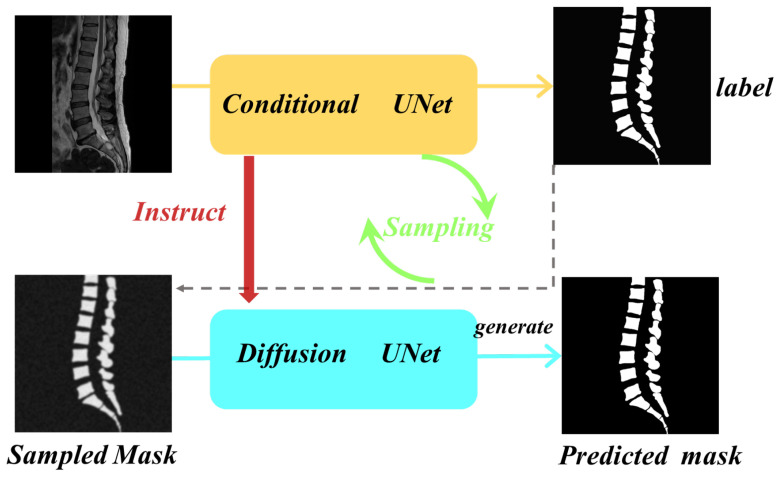
Modeling conceptual diagram. The diffusion model alternates between the noise addition phase and the sampling phase to train the network and ultimately generate the final prediction mask. The conditional U-Net model induces the diffusion model to synergize the advantages of the existing discriminative segmentation and generative diffusion models.

In this study, we built upon the foundation of diffusion models and cleverly integrated conditional models to provide more refined feature guidance. This approach achieves a deep combination of the advantages of existing discriminative segmentation models and generative diffusion probabilistic models. Our objective is to explore an efficient and high-quality method for precise segmentation of spinal multi-modal images. To this end, we propose a conditional diffusion segmentation framework named Verdiff-Net (as illustrated in Figure 2, which shows a conceptual diagram of our proposed segmentation framework). We observed that conventional diffusion segmentation models often adopt a simple concatenation approach when learning and modeling the joint probability of features from the original image and segmentation mask. To overcome this limitation, we introduced a multi-scale fusion module in the conditional U-Net to comprehensively capture the feature information of the original image. Additionally, by ingeniously applying the noise semantic adapter, we filtered out irrelevant information, allowing the model to focus more on the vertebral regions, thereby effectively enhancing the fineness of the segmentation masks and overall performance.

The innovations and contributions of this study can be summarized as follows:

(1) Synergistic combination of models: Verdiff-Net effectively blends the advantages of diffusion and conditional models, improving segmentation tasks’ accuracy and stability, especially when modeling discrete targets.
(2) Multi-scale fusion module: Verdiff-Net integrates a multi-scale fusion module into the conditional U-Net to overcome the drawbacks of traditional diffusion models. By efficiently capturing and maintaining the underlying spinal feature information, this module lessens the loss that is commonly brought about by coarsely fusing image and mask features.
(3) Noise semantic adapter (NSA): During the diffusion process, the NSA serves as a selective filter, drawing the model’s attention to important spinal aspects while rejecting unimportant data. This breakthrough increases inference efficiency as well as the model’s accuracy in spinal segmentation.
(4) Comprehensive evaluation: The effectiveness and generalizability of the model are validated on four vertebrae medical datasets with three different modalities. To the best of our knowledge, this study is the first to thoroughly evaluate the model’s robustness and generalization on a multi-modality vertebrae dataset.

## 2. Methodology

### 2.1. Framework Overview

Our proposed Verdiff framework consists of two key components: Conditional U-Net and diffusion U-Net. The core idea is to combine the discriminative power of existing segmentation models with the generative capabilities of diffusion models to enhance medical image segmentation. The conditional U-Net provides a prior segmentation mask for the diffusion model, while the diffusion U-Net iteratively refines the segmentation mask in an effective, efficient, and interactive manner.

In this section, we briefly introduce the proposed diffusion model framework. Unlike traditional medical image segmentation methods that directly input raw image data to predict the corresponding segmentation label map, the prior mask f(b) generated by the conditional U-Net undergoes iterative refinement through the diffusion U-Net, ultimately producing the final segmentation mask. During the forward diffusion process, the prior mask is used for noise addition, and during the reverse diffusion process, it serves as the initial sampling point. The entire diffusion process can be represented as follows:

(1) $$x_{T}⇌\cdots ⇌x_{t}\overset{p_{\theta }\left(x_{t-1}|x_{t},\;f\left(b\right)\right)}{\underset{q(x_{t}|x_{t-1},\;f\left(b\right))}{⇌}}x_{t-1}⇌\cdots ⇌x_{0}$$

**Figure 3:**
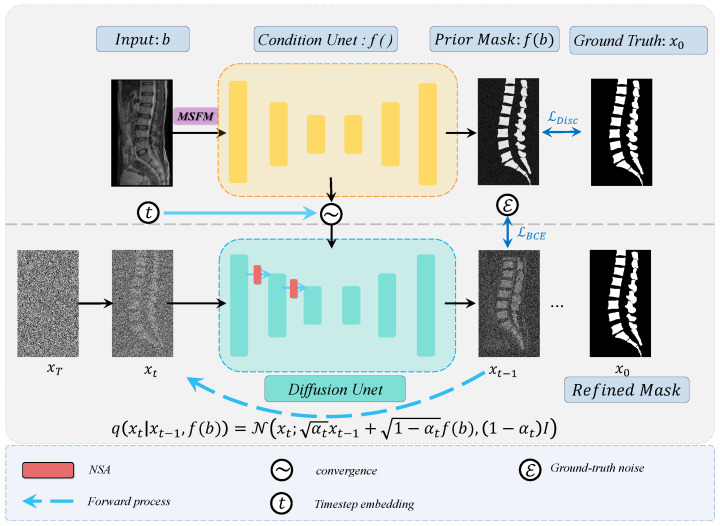
Overview of the Verdiff-Net framework. It contains two different U-Net backbone encoder–decoder structures.

The diffusion model takes noisy segmentation labels $x_{t}$ and the original image b as input and learns spinal features under the guidance of prior information from the original image. It predicts clear mask results through a reverse denoising process. Figure 3 illustrates the overall architecture of the proposed network, which consists of a forward diffusion stage and a reverse stage. Next, we provide a detailed explanation of the two key modules: the multi-scale fusion module (MSFM) and the noise semantic adapter (NSA).

### 2.2. Training Strategies for Diffusion Models

#### 2.2.1. Diffusion Forward Process

During the training phase, variational inference is performed over a Markov process with *T* time steps. In the forward process at each time step t∈T, successive Gaussian noise is added to the label $x_{0}$ until the image becomes pure isotropic Gaussian noise $x_{T}$, thereby learning the data distribution. To better guide the diffusion process, we introduce an initial segmentation mask f(x) provided by the conditional U-Net f(b), which is used to correct the noise process. The forward noise process in the Markov chain at time step *t* in the forward diffusion process is represented as follows:

(2) $$q\left(x_{t}|x_{t-1},f\left(b\right)\right)=N\left(x_{t};\sqrt{\alpha_{t}}x_{t-1}+\sqrt{1-\alpha_{t}}f\left(b\right),\left(1-\alpha_{t}\right)I\right),$$

where N(·) denotes a normal distribution with a mean of $\alpha_{t}x_{t-1}+\left(1-\alpha_{t}\right)f\left(x\right)$, and a variance of $(1-\alpha_{t})I$. Here, f(x) represents the segmentation prior generated by the discriminative model, which is used to correct the noise distribution at each time step, allowing Gaussian noise to better align with the segmentation prior. The parameter $\alpha_{t}$ controls the noise variance and determines the amount of noise added. $\left(x_{0},x_{1},\ldots ,x_{T}\right)$ denotes the *T* steps in the Markov chain, and *I* is the identity matrix, representing isotropic noise.

After *T* steps, the data transform into a pure Gaussian noise image, represented by $x_{T}$, and we can establish the relationship between $x_{0}$ and $x_{t}$ as follows:

(3) $$x_{t}=\sqrt{{\bar{\alpha }}_{t}}x_{0}+\sqrt{1-{\bar{\alpha }}_{t}}f\left(b\right)+\sqrt{1-{\bar{\alpha }}_{t}}\epsilon$$

where ${\bar{\alpha }}_{t}=\prod_{s=1}^{t}\alpha_{s}$ is the cumulative noise scheduling, which controls the degree of interpolation between $x_{0}$ and the noise. The term f(b) is the output of the conditional U-Net, which helps to guide the noise interpolation process. The term ϵ∼N(0,I) represents standard Gaussian noise.

During the training process, after obtaining the noisy label $x_{t}$ at time step *t*, the objective is to predict a clear label map $x_{0}$ during the sampling phase. By combining the prior information from the conditional U-Net f(b), the generative model can more efficiently recover the segmentation mask $x_{0}$ from the noise, while further optimizing the synergy between the discriminative model and the generative model throughout the learning process.

#### 2.2.2. Diffusion Reverse Process

Where $\bar{\alpha }$ is the cumulative product of α, used to simplify the relationship formula from the initial time step to the current time step. After obtaining the noisy label $x_{t}$ at time step *t*, our goal is to predict the clean label map $x_{0}$ at the sampling stage. The holistic network $\Phi_{\theta }$ uses the knowledge gained from the forward process to generate a sequence of incremental denoising operations in the reverse denoising process, which yields a clear image. The reverse distribution, as shown in Equation (4), reduces to a problem of minimizing the KL divergence between the forward and reverse distributions for all time steps.

(4) $$p\left(x_{t-1}|x_{t},f\left(b\right)\right):=N\left(x_{t-1};\mu_{\theta }\left(x_{t},t,f\left(b\right)\right),\Sigma_{\theta }\left(x_{t},t,f\left(b\right)\right)\right)$$

where $x_{t-1}$ follows a normal distribution (Gaussian distribution) with its mean and variance determined by the parameterized functions $\mu_{\theta }$ and $\Sigma_{\theta }$. $\mu_{\theta }$ is the conditional mean function, and $\Sigma_{\theta }$ is the conditional variance (covariance) function. The optimization of the overall function requires sampling from the distribution $q(x_{t}|x_{t-1})$. Given $x_{0}$, the marginal distribution of $x_{t}$ can be obtained from the intermediate latent variables.

(5) $$q\left(x_{t}|x_{0},f\left(b\right)\right)=N\left(x_{t};\sqrt{{\bar{\alpha }}_{t}}x_{0}+\sqrt{1-{\bar{\alpha }}_{t}}f\left(b\right),\left(1-{\bar{\alpha }}_{t}\right)I\right)$$

By utilizing the posterior distribution $q(x_{t}|x_{t-1},x_{0})$, the KL divergence between the forward and reverse distributions is further simplified and reduced. Under the Markov assumption, the posterior distribution $q(x_{t-1}|x_{t},x_{0})$ is obtained through the iterative denoising process at each time step, yielding the estimate of $x_{t-1}$.

(6) $$q\left(x_{t-1}|x_{t},x_{0},f\left(b\right)\right)=N\left(x_{t-1};\mu \left(x_{t},x_{0},f\left(b\right)\right),\sigma^{2}I\right)$$

where

$$\mu \left(x_{t},x_{0},f\left(b\right)\right)=\frac{\sqrt{{\bar{\alpha }}_{t-1}}\left(1-\alpha_{t}\right)}{1-{\bar{\alpha }}_{t}}x_{0}+\frac{\sqrt{\alpha_{t}}\left(1-{\bar{\alpha }}_{t-1}\right)}{1-{\bar{\alpha }}_{t}}x_{t}+\sqrt{1-{\bar{\alpha }}_{t}}f\left(b\right)$$

and $\sigma^{2}=\frac{\left(1-{\bar{\alpha }}_{t-1}\right)\left(1-\alpha_{t}\right)}{1-{\bar{\alpha }}_{t}}$. Assuming that the covariance matrices of the two distributions $q(x_{t-1}|x_{t},x_{0})$ and $p(x_{t-1}|x_{t})$ are the same, the corresponding expression of $x_{t-1}$ as shown in Equation (7) is further obtained by the iterative inference process of the denoising model.

(7) $$x_{t-1}\leftarrow \frac{1}{\sqrt{\alpha_{t}}}\left(x_{t}-\frac{1-\alpha_{t}}{\sqrt{1-{\bar{\alpha }}_{t}}}\Phi_{\theta }\left(x_{t},t,f\left(b\right)\right)\right)+\sigma_{t}z$$

where z∼N(0,I), t=1,…,T, σ is the inverse variance that can be learned, and *z* is the random sampling parameter. According to the above Equations (4)–(6), the simplified training objective is finally obtained by the following:

(8) $$L_{\mathrm{simple}}=E_{x_{0},\epsilon }\left[∥\epsilon -\Phi_{\theta }\left(\tilde{x},t,f\left(b\right)\right){∥}_{2}^{2}\right]$$

where ϵ∼N(0,1). We model the medical image segmentation task as a discrete data generation problem and directly predict the expected value of a label $x_{t}$ with noise. Based on the variational upper bound of the negative log-likelihood of previous diffusion models, we use the Kullback–Leibler (KL) divergence and the binary cross entropy (BCE) mixed loss. Based on the combining Equations (6) and (10), we obtain the total hybrid loss [22] by the following:

(9) $$L_{\mathrm{BCE}}=-E_{\left(\epsilon ,\hat{\epsilon }\right)}\sum_{i,j}^{H,W}\left[\epsilon_{i,j}\log{\hat{\epsilon }}_{i,j}+\left(1-\epsilon_{i,j}\right)\log\left(1-{\hat{\epsilon }}_{i,j}\right)\right]$$

(10) $$L_{\mathrm{total}}=L_{\mathrm{simple}}+L_{\mathrm{BCE}}$$

By taking the average of different predicted segmentation masks generated by the network $\Phi_{\theta }$, a clear mask of significance can finally be obtained, providing a valuable reference for radiologists. The flow of the algorithm is shown in Algorithms 1 and 2.
**Algorithm** **1:** Training1. $\left(b,x_{0}\right)∼q\left(b,x_{0}\right)$2. T∼Uniform({1,2,…,T})3. ϵ∼N(0,I)4. **Calculate Equation** (5)5. **Take** gradient descent on $\nabla_{\theta }\left(L_{\mathrm{total}}\right)$6. **until** converged

**Algorithm** **2:** Sampling
1. $x_{T}∼N\left(0,I\right)$
2. **for** t=T,…,1 **do**
3. z∼N(0,1) if t>1, else z=0
4. $x_{t-1}=\frac{1}{\sqrt{\alpha_{t}}}\left(x_{t}-\frac{1-\alpha_{t}}{\sqrt{1-{\bar{\alpha }}_{t}}}\Phi_{\theta }\left(x_{t},t,f\left(b\right)\right)\right)+\sigma_{t}z$
5. **end for**
6. **return** $x_{0}$


### 2.3. Multi-Scale Fusion Module (MSFM)

**Figure 4:**
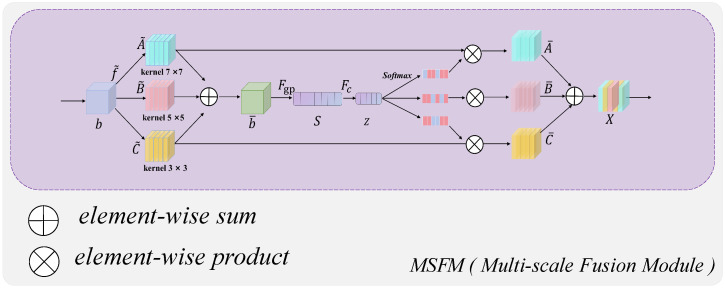
Schematic structure of the multi-scale fusion module (MSFM).

Conditional diffusion-based probabilistic models introduce noise into the original label and iteratively predict the segmented label map. However, the noise-added mask information at the current time step is often coarsely spliced and processed with the original image during fusion, which undoubtedly leads to feature loss and degrades the capability of subsequent network segmentation. To solve this problem, the proposed MSFM will play a role. As shown in Figure 4, before the input raw image *b* enters the conditional U-Net coding and decoding structure of the model, we first transform the raw data $b\in R^{H\times W\times C}$ by three different convolutional kernel sizes of 3×3, 5×5, and 7×7, and fuse the results of the three branches by element summation to obtain $\tilde{b}$.

(11) $$\tilde{b}=\tilde{A}+\tilde{B}+\tilde{C}$$

where $\tilde{b}\in R^{H\times W\times C}$. After generating the channel information, the global information is embedded by using global average pooling to obtain $S\in R^{C}$. The *n*-th element of *S* is obtained through contracting $\bar{b}$ by using the spatial dimension H×W.

(12) $$s_{n}=F_{gp}\left(\bar{b}\right)=\frac{1}{H\times W}\sum_{i=1}^{H}\sum_{j=1}^{W}{\left(\bar{b}\right)}_{n}\left(i,j\right)$$

Specifically, we use the ELU activation function in the fully connected layer along with the batch normalization technique for fine-tuning. To filter out useless dimensions to improve efficiency, we use the fully connected layer to create a compressed feature $z\in R^{d}$ to achieve an accurate and adaptive selection guide.

(13) $$z=Fc(\bar{b})$$

where *d* is a hyperparameter that can be fine-tuned, it is used to control the dimensionality of the compressed feature *z*.

Then, cross-channel soft attention is used to adaptively select information at different spatial scales. Where *a*, *b*, *c* denote the soft attention vectors of $\tilde{A}$, $\tilde{B}$, $\tilde{C}$, respectively, and $a_{n}$ is the n-th element of *a* and $a_{n}+b_{n}+c_{n}=1$, $A,B,C\in R^{C\times d}$. Finally, the final feature map *X* is obtained from the attention weights on each convolutional kernel:

(14) $$X_{n}=a_{n}\cdot {\tilde{A}}_{n}+b_{n}\cdot {\tilde{B}}_{n}+c_{n}\cdot {\tilde{C}}_{n},$$

where $X=[X_{1},X_{2},\ldots ,X_{n}]$, $X_{n}\in R^{H\times W}$,

$$\begin{array}{cc} a_{n} & =\frac{e^{A_{n}z}}{e^{A_{n}z}+e^{B_{n}z}+e^{C_{n}z}},\\ b_{n} & =\frac{e^{B_{n}z}}{e^{A_{n}z}+e^{B_{n}z}+e^{C_{n}z}},\\ c_{n} & =\frac{e^{C_{n}z}}{e^{A_{n}z}+e^{B_{n}z}+e^{C_{n}z}}. \end{array}$$

The output of *X* from this fusion module is then refined to extract the segmentation features of the original image, being fused with the information from the current step’s noise-added mask $x_{t}$, and finally input to the U-Net coding structure of the diffusion model.

### 2.4. Noise Semantic Adapter (NSA)

Spinal pictures are used in anatomy to illustrate very intricate systems such as the spinal cord, IVD, and vertebrae. Not only are these structures connected, but they are also all extremely intricate. A continuous succession of structurally similar but individually unique vertebrae make up the vertebrae. The vertebrae are distinguished by their symmetry and continuity, which may not be as noticeable in other kinds of medical pictures. Vertebrae images from current medical imaging methods frequently have high aspect ratios, which makes processing image size and feature extraction difficult for popular deep learning networks. To further complicate the picture segmentation process, the key target regions of the vertebrae are typically more concentrated than in other medical images, and there is a certain semantic coherence between these regions. Relatively speaking, if researchers choose to compress the original vertebrae image into a square, or resize the original image to make it easier to input into the model, these operations will undoubtedly cause great damage to the spatial information features of the original image and lead to semantic loss. For example, in a single step in the diffusion process, *t*, the U-Net decoder of the diffusion model receives the fusion information from the noise mask, $x_{t}$, and the original image, and finally predicts the resultant clear mask. Along with input of different types of medical images, the lack of the model’s ability (namely, to extract semantic information from a particular image) can lead to large discrepancies in the model’s predictions.

**Figure 5:**
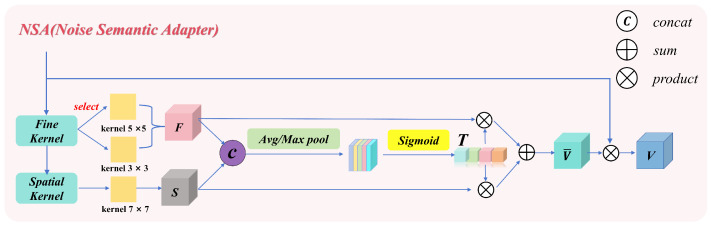
Noise semantic adapter (NSA) structure.

To overcome this negative effect, we adopted the backbone structure of MedSegDiff [20]. The NSA was introduced into the standard U-Net of the conditional model to extract the spinal semantic information from the fused features. Specifically, we introduce the NSA into the U-Net encoder of the diffusion model. As shown in Figure 5, compared to the standard 2D convolution embedded in the standard U-Net, the NSA provides an operation to automatically select the convolution kernel. The input U-Net feature image information $X\in R^{H\times W\times C}$ is first operated by the initial sliding window (kernel operation of the fine-grain convolution layer), and then by the spatial convolution layer of the 7 × 7 convolution kernel. The corresponding matrix obtained by these two operations is *F* and *S*, where $F,S\in R^{H\times W\times C/2}$. The first fine-grained convolution layer can be selected with a 3 × 3 or 5 × 5 convolution layer, keeping the number of channels constant. Here, note that the specific selection strategy (for the convolution kernel geometry size) depends on the number of mask pixels around the center pixel. For fine particle extraction, 3 × 3 volume nodes are used when processing pixel data within the vertebrae, including vertebrae boundaries. For extracting large area background feature information, a 5 × 5 volume combination with a padding of 2 is used for coarse extraction, which improves the model’s degree of matching to the target region features. In the next spatial convolution layer, a larger 7 × 7 convolution kernel with a dilation of 3 is employed. This approach increases the receptive field without adding more parameters, and each input channel is convolved with its own set of filters.

The two convolution layers are used to halve the dimension of the channel, and the obtained results are concatenated by aggregating the average and maximum attention. The sigmoid function is then applied to smoothly activate the weighted attention, attempting to expand the channel dimension back to its original size to retain the most relevant information and obtain the detailed features *T* of each channel.

(15) T=Sigmoid·f(F∗S)

where ∗ represents the product, $T\in R^{H_{1}\times W_{1}\times C}$.

The two convolution layers are used to halve the dimension of the channel, and the obtained results are concatenated by aggregating the average and maximum attention, and the sigmoid function is applied to smoothly activate the weighted attention, trying to expand the channel dimension back to its original size to retain the most relevant information and obtain the detailed features *T* of each channel.

After multiplying and superimposing the matrices *F* and *S* obtained from two scale convolution layers, $\bar{V}\in R^{H\times W\times C}$ is then obtained. The final output feature $V\in R^{H\times W\times C}$ results from multiplying with the residual connection of the original input information *X*. The *V* can be expressed as follows:

(16) $$V=\bar{V}*X,\bar{V}=F*T+S*T$$

## 3. Experiments and Results

### 3.1. Dataset Introduction

To validate the generalizability and robustness of the proposed model, we comprehensively evaluated our proposed model on four vertebrae imaging dataset, including two sets of CT datasets, respectively, from publicly and privately available ones, a single set of MR ones, and a single set of X-ray ones.

**Public CT dataset.** We used the 42 sets of 3D spine1K data in the “verse” folder of the large-scale spine CT dataset called CTSpine1K. Based on this dataset, we conducted vertebrae segmentation experiments to select slices and contacted radiologists to manually select images one by one, and finally selected 2508 images with clear layers and anatomical information with masks for training and sampling. All the image sizes are standardized to 166 × 369.
**Private CT dataset.** Sagittal CT images of the vertebrae were collected from 630 volunteers (396 females, 234 males; mean age 26 ± 3 years, range 19 to 36 years). Three radiologists and one vertebral surgeon labeled these vertebral image regions as the ground truth for the vertebrae segmentation task and checked the labeled regions against each other to ensure reliability. Thus, each subject had a T2-weighted MR image and a corresponding mask as the initial ground truth, where each vertebra was assigned a unique label. We chose images of clean lumbar vertebral regions containing the caudal vertebrae and standardized the dimensions to 534 × 768 as inputs.
**Private MR dataset.** The dataset, collected from a local hospital, consists of T2-weighted MR volumetric images from 215 subjects. Among the 215 subjects, there were 6 normal subjects, 177 patients with vertebrae degeneration (VD), 204 patients with intervertebral disk degeneration (IDD), 21 patients with lumbar spondylolisthesis (LS), 91 patients with spinal canal stenosis (SCS), 22 patients with Schmorl’s node (SN), and 53 patients with vertebral endplate osteochondritis (VEO). The delineated mask was corrected by the senior expert using the ITK-SNAP1 to be the ground truth of vertebrae parsing [26]. The average pixel spacing within the plane of the MR image was 0.35 mm, while the average slice thickness was 4.42 mm. By focusing solely on segmenting the vertebrae and excluding the spinal fluid portion between them, we first removed the spinal fluid from the labels of these data. We then smoothly extracted the 3D raw data and their corresponding mask data from the sagittal plane, ultimately obtaining pure 880 × 880 paired images and mask.
**Public X-ray dataset.** The dataset consists of 609 spinal anterior–posterior X-ray images [27]. The landmarks were provided by two professional doctors at the London Health Sciences Center. Each vertebra was located by four landmarks with respect to four corners. All the image sizes were 250 × 750. In addition, since the noise artifacts in this dataset were extremely severe, and some of the data were mixed with medical clinical instruments on the vertebrae, in order to further test the segmentation ability of the model in this particular condition, we ignored the detailed features of the vertebrae contour lines and performed a binary transform only for the vertebral trunk regions in order to obtain the original annotations.

### 3.2. Data Preprocessing

We used dynamic data augmentation during training to avoid network overfitting and to increase the robustness of our model. Three types of data augmentation are included, as follows: (1) randomly rotating the image from −30° to 30° to simulate the rotation variance; (2) randomly shifting the image by 1–5% to simulate the shift variance; (3) random elastic deformation and random contrast adjustment to improve the generalization of the model. To ensure all the methods used the same augmented training data, we used the same random state (seed = 35) for all methods when conducting data augmentation.

### 3.3. Training/Validation Setup and Evaluation Metrics

All the networks were built using PyTorch on a Linux 20.04 system, and the code ran on a server equipped with an A6000 GPU unless otherwise specified. The training process had a maximum iteration limit of 9000 steps. In our implementation, we trained our networks from scratch without relying on any pre-trained models. We employed the Adam optimization algorithm to minimize the loss function as described in Equation (9). Given our computational resources, a batch size of 1 was chosen. Batch normalization was utilized to facilitate a higher learning rate, resulting in relatively shorter training times. The learning rate was set to $10^{-4}$. The stopping criterion was determined by the point where the validation loss stopped decreasing.

The Dice similarity coefficient (DSC) and intersection over union (IoU) serve as quantitative metrics for assessing segmentation performance. The DSC quantifies the overlap between the ground truth segmentation (Y) and the predicted segmentation results (X). The IoU describes the ratio of the overlapping area to the union area between the predicted and annotated segmentation regions. Specifically, the IoU is calculated as the area of intersection divided by the area of union between the predicted and annotated regions.

(17) $$\mathrm{Dice}=\frac{2|X\cap Y|}{\left|X|+|Y\right|}$$

(18) $$\mathrm{IoU}=\frac{\mathrm{TP}}{\mathrm{TP}+\mathrm{FP}+\mathrm{FN}}$$

Here, TP is the number of true positive pixels, which are correctly identified as part of the segment of interest. FP is the number of false positive pixels, representing the count of pixels incorrectly classified as part of the segment. FN is the number of false negative pixels, which are the pixels that are part of the segment in the ground truth but were missed by the prediction.

### 3.4. Vertebrae Segmentation Results

**Figure 6:**
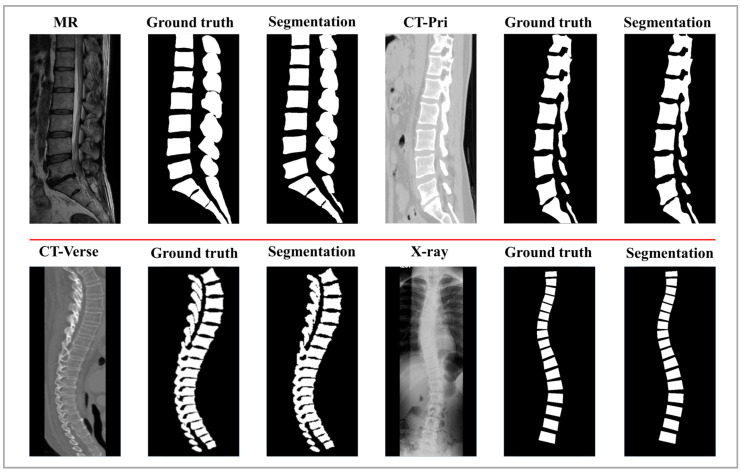
Verdiff-Net achieves reliable spinal segmentation performance. Above are the midsagittal sections of the vertebrae of each of the four subjects. The four subjects were imaged using different vertebrae imaging devices.

To quantitatively evaluate Verdiff-Net, we used Dice and IoU to measure the difference between the predicted results and the ground truth. During the evaluation, the ground truth labels for the vertebrae were obtained by manually drawing lines along the vertebral contours and were confirmed by certified radiologists. Verdiff-Net achieved average Dice scores of 94.37 ± 4.75%, 93.84 ± 1.56%, 93.86 ± 1.98%, and 88.74 ± 21.6% for vertebral structure segmentation on four different spinal multi-modality datasets. These quantitative results indicate that Verdiff-Net effectively balances over-segmentation and under-segmentation. As shown in Figure 6, we visualized the segmentation results of a single sagittal slice of the lumbar vertebrae. The high similarity between the model-predicted masks and the ground truth masks demonstrates that the model successfully achieves precise 2D spinal image segmentation across four multi-modality datasets.

### 3.5. Comparison of the Results

In a comparative evaluation across four multi-modality datasets, we compared our proposed segmentation method with state-of-the-art deep learning approaches, as detailed in Table 1. We evaluate our approach against a number of cutting-edge segmentation approaches, such as U-Net [28], FCN [29], DeepLabv3 [30], nnU-Net [31], and Swin-U-Net [12]. For a thorough comparison, we additionally take into account the most recent state-of-the-art methods, SAM [32], and MedSegDiff [20].

**Figure 7:**
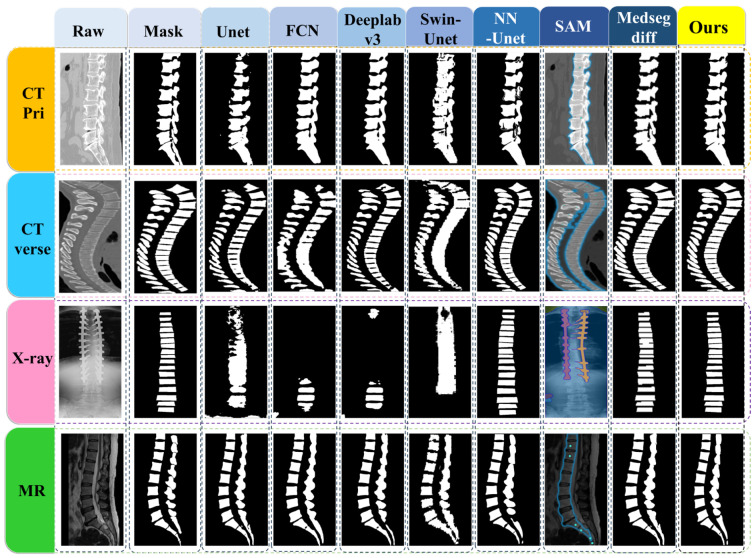
The comparison of Verdiff-Net with several SOTA segmentation methods.

All experiments were conducted on the same datasets, and statistical significance was noted in the results. The results show that Verdiff-Net performs better than its rivals, attaining the highest Dice similarity in the datasets CT-Pri, CT-Verse, and MR. More specifically, on the MR public dataset, Verdiff-Net demonstrated a considerable Dice improvement of roughly 1.7% over nnU-Net, and improvements of 0.51% and 1.4% on CT-Pri and CT-Verse, respectively. Verdiff-Net demonstrated its supremacy by routinely delivering Dice scores above 93% on CT-Pri, CT-Verse, and MR. Verdiff-Net reduces false positives and improves Dice and IoU scores by incorporating the suggested module and taking advantage of diffusion models’ pixel-level semantic segmentation capabilities. Verdiff-Net generates smoother and more accurate vertebral mask edges with a considerable reduction in pixel shifts and misclassifications as compared to other DDPM-based techniques such as MedSegDiff [20]. Figure 7 makes clear that alternative deep learning techniques are not able to reliably produce accurate spinal segmentation in all datasets, especially when it comes to X-ray data, where Dice coefficients for U-Net, FCN, Swin-U-Net, and SAM were less than 70%. In regions above the implant, FCN and DeepLabv3 are unable to segment the vertebrae; in contrast, nnU-Net, MedSegDiff, and our suggested model can recognize and find the vertebrae.

In conclusion, Verdiff-Net outperforms other techniques in terms of Dice similarity and robustness to alterations in input modalities, demonstrating its strength in delivering accurate spinal segmentation, even in difficult settings.

### 3.6. Ablation Study

We conducted a thorough ablation experiment on the MR dataset to confirm the efficacy of our proposed module. To assess how well the three activities were performed, we used dice scores (%). The segmentation results of the underlying denoising diffusion probabilistic model (DDPM) on the MR dataset are greatly improved by both the Noise semantic adapter (NSA) alone and the multi-scale fusion module (MSFM) module, as shown in Table 2. In particular, the model’s Dice coefficient is improved by 3.28% just by using the suggested multi-scale fusion module (MSFM) in place of the earlier technique.

This shows that by helping to retrieve the original vertebrae image’s finely segmented features, which can then be used to include semantic requirements in the diffusion U-Net model that follows, multi-scale fusion module (MSFM) can optimize the DDPM-based model. By learning an architecture based on the content-switching convolutional kernel of the original vertebrae images, the noise semantic adapter (NSA) improves the interaction between the noise mask and the anatomical semantic features of the images. This greatly improves the segmentation results of the underlying diffusion model, leading to an improvement of the Dice coefficients by 1.59%, respectively. In this MR dataset, our suggested two modules enhance the base denoising diffusion probabilistic model by over 5%, yielding cutting-edge segmentation outcomes. The ablation study’s findings confirm the efficacy of the suggested modules by demonstrating that the semantic feature extractor is responsible for the notable increase in vertebrae parsing performance.

## 4. Discussion

### 4.1. Segmentation Effects

**Figure 8:**
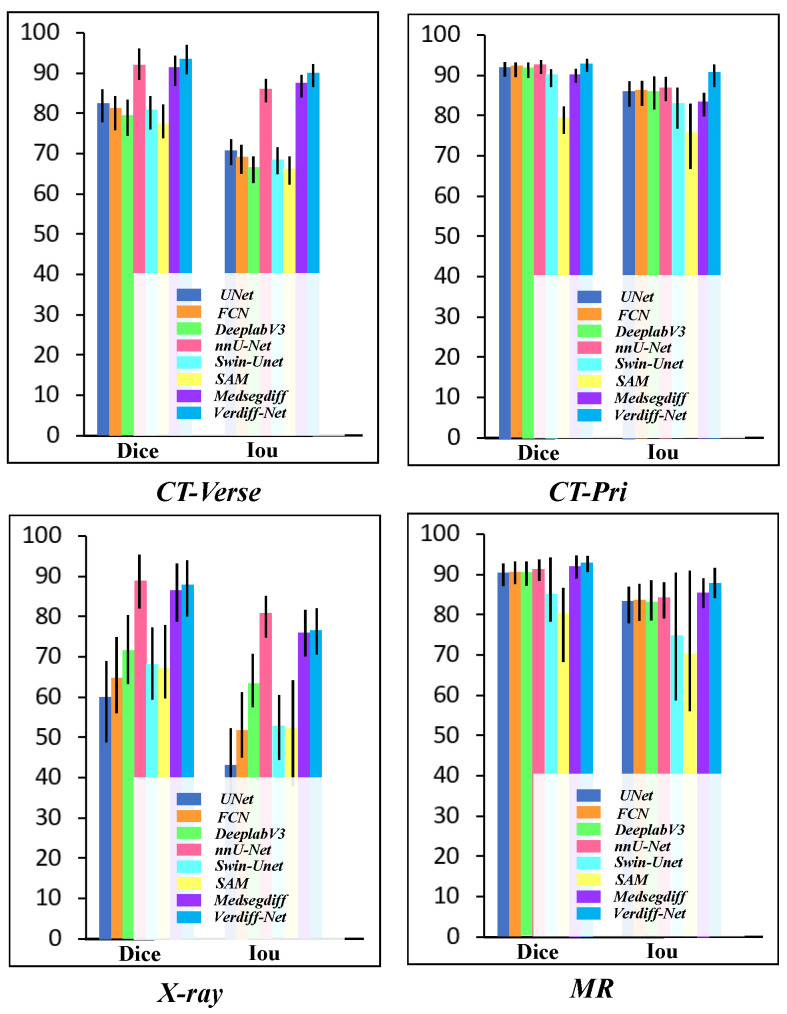
Vertebrae segmentation metrics (Dice vs. IoU) on the four datasets. In the majority of vertebrae datasets, Verdiff-Net achieved the highest average Dice (%) results.

We analyzed the segmentation effects across four disparate datasets. The comparative trials revealed significant variations in the models’ performance, as depicted in Figure 8. Specifically, on the CT-Verse and CT-Pri datasets, the models exhibited superior segmentation capabilities, accompanied by a notably smaller total standard deviation. In contrast, the X-ray dataset presented a challenge, with the segmentation metrics Dice and IoU recording lower values and the overall standard deviation being slightly elevated. To elucidate these discrepancies, we undertook an exhaustive review of the dataset composition. Less overall variability of the pictures in the dataset was caused by the subject’s mid-sagittal vertebrae segment data in the CT dataset being smoother and having higher resolution. On the other hand, the X-ray dataset’s poor segmentation can be attributed to two key factors. Firstly, there is a lot of noise and artifacts in the X-ray collection, and the vertebrae shape differs a lot from patient to patient, making segmentation more challenging. Second, with no discernible edge enhancement or description, the target vertebrae region is remarkably similar to the background pixels. Many of the most advanced segmentation methods struggle to handle this high degree of similarity, making it simple to confuse the vertebrae region with the backdrop. Furthermore, our observations indicate that the standard deviation of the SAM model’s segmentation outcomes on the MR dataset is significantly higher when compared to other models, including Swin-U-Net. The spinous processes in the MR images exhibit substantially lower resolution and brightness compared to the vertebral trunk, often blending in with the background. Additionally, the manually annotated masks for these areas are less regular, contrasting with the more uniform imaging conditions typical of CT scans. In this context, the absence of pre-training hinders the SAM model’s ability to effectively segment the vertebral feature region from a semantic standpoint, thereby reducing the overall segmentation accuracy of the dataset.

Due to Swin-U-Net’s relatively slow global sensitivity and convergence rate during actual training, its segmentation performance is further compromised by the system’s limited capability to extract meaningful features from the vertebral region. The performance of these models on the MR dataset also highlights their limitations in dealing with low-resolution images of vertebrae that have a background highly similar to the foreground, underscoring the need for more sophisticated models that can better handle such challenging imaging conditions.

Furthermore, we examined the instances in which certain models failed the comparison test, as illustrated in Figure 7. Of the models tested on the X-ray dataset, only nnU-Net, MedSegDiff, and Verdiff-Net were able to accurately predict the specific regions that belong to the vertebrae in the X-ray images when bone nails were present, and they also maintained a high degree of similarity with the mask that the qualified physicians had labeled. Based on our analysis, MedSegDiff and Verdiff-Net’s segmentation success can be attributed to distinct factors. Specifically, the DDPM algorithm, which emphasizes learning only the vertebrae region’s specific features during the noise addition process, disregards the features of the other parts as noise. By classifying all non-vertebrae pixels on the vertebrae layer as noise during the reverse denoising stage, these models successfully segment the vertebrae area. On the other hand, nnU-Net’s integrated data preparation module improves the vertebrae region’s semantic information and sensory field, which helps the network better collect vertebrae properties.

### 4.2. Limitation

From an application perspective, we acknowledge that we only considered the segmentation of spinal images and did not evaluate its generalizability to other medical images. Additionally, due to the difficulty in obtaining and annotating data, there are differences in the spinal regions (such as thoracic and lumbar spine) among different datasets, and the annotation methods vary. The imaging techniques for different spinal regions also lead to variations in imaging directions, resulting in different morphological representations of the spine. This not only increases the difficulty of data annotation but also complicates the model’s ability to recognize vertebral regions.

On the one hand, our initial intention was to explore the generalization performance of the diffusion model on multi-modal spinal images and the stability of segmentation performance across different modality datasets. This led us to overlook the model’s multi-class segmentation performance, simplifying the spinal segmentation task to a binary segmentation task. On the other hand, the segmentation task for the generative model requires significant CUDA memory, thereby increasing computational demands. Segmenting multiple types of spinal regions (≥9 categories) inevitably leads to high CUDA usage, potentially causing errors.

Despite the algorithm’s superior segmentation performance, there are still shortcomings. First, the DPM-based algorithm takes a long time to train. Due to the necessity of performing addition and denoising operations over several iterations, the training process is time-consuming. Secondly, this approach heavily relies on GPU resources. The training phase requires handling a large number of computational operations, typically necessitating high-performance GPU support, which limits its applicability to some extent. It is recommended that future research focus on optimizing the algorithm, enhancing training efficiency, and reducing dependence on hardware resources to further promote the application of this technology in clinical settings.

## 5. Conclusions

This study proposes a novel medical image segmentation method called Verdiff-Net, which combines the advantages of discriminative segmentation models and generative diffusion probabilistic models to achieve accurate segmentation of multi-modal spinal images. The contributions of this study are summarized as follows:

**Feature extraction mechanism**: A feature extraction mechanism incorporating multi-scale convolutional layers is introduced into the conditional U-Net. This mechanism reduces the loss of low-level features, effectively models the underlying data distribution, and maximizes the model’s ability to learn multi-scale spatial features from the original spinal images.

**Noise semantic adapter (NSA)**: Given the characteristic large aspect ratio of spinal medical images, NSA is proposed. NSA filters the fused features input into the diffusion model and further adjusts the model to focus on attention feature responses in the spinal target area, thus accommodating the unique morphology and structural factors of the spine.

**Validation and generalization capability**: The effectiveness and generalization capability of the model were thoroughly evaluated on four medical datasets covering three different imaging modalities. The results demonstrate that Verdiff-Net exhibits outstanding performance in the domain of spinal medical image segmentation, effectively identifying and segmenting low-level spinal features while showing strong potential for application across different datasets. This study is the first to comprehensively evaluate the robustness and generalization capability of the model on multi-modal spinal datasets, providing new perspectives and references for research in this field, and promoting further exploration and development in medical image segmentation technology.

## Figures and Tables

**Table 1:** Metrics unique to each experiment for the four datasets.

	CT-Verse		CT-Pri		X-ray		MR	
	Dice (%)	IoU (%)	Dice (%)	IoU (%)	Dice (%)	IoU (%)	Dice (%)	IoU (%)
U-Net [28]	83.25 ± 4.08	71.35 ± 3.23	93.22 ± 1.83	87.34 ± 3.19	60.78 ± 10.23	43.75 ± 9.82	91.23 ± 2.76	84.00 ± 4.59
FCN [29]	82.22 ± 4.34	69.88 ± 3.61	93.24 ± 1.79	87.36 ± 3.07	65.30 ± 9.53	52.60 ± 8.17	91.50 ± 2.75	84.44 ± 4.57
DeepLabv3 [30]	80.41 ± 4.50	67.29 ± 3.27	93.06 ± 1.88	86.94 ± 4.05	72.44 ± 8.59	63.91 ± 6.72	91.06 ± 2.93	83.70 ± 5.15
nnU-Net [31]	92.98 ± 3.86	86.97 ± 2.93	93.33 ± 1.69	88.04 ± 2.97	**89.77 ± 6.69**	**81.69 ± 5.26**	92.16 ± 2.73	84.97 ± 4.53
Swin-U-Net [12]	81.65 ± 4.19	69.27 ± 3.44	91.27 ± 2.15	84.02 ± 5.64	68.93 ± 9.06	53.13 ± 8.08	85.86 ± 7.94	75.39 ± 16.48
SAM [32]	79.78 ± 4.23	66.89 ± 3.50	80.36 ± 3.44	76.35 ± 8.21	67.89 ± 9.22	52.56 ± 13.32	81.00 ± 9.27	70.92 ± 17.57
MedSegDiff [20]	92.44 ± 3.75	88.27 ± 2.91	91.07 ± 1.69	84.51 ± 2.86	87.59 ± 7.24	76.68 ± 5.78	92.96 ± 2.87	86.53 ± 3.71
Verdiff-Net(ours)	**94.37 ± 3.73**	**90.89 ± 2.87**	**93.84 ± 1.56**	**91.87 ± 2.79**	88.74 ± 7.04	77.04 ± 5.75	**93.86 ± 1.98**	**88.58 ± 3.83**

**Table 2:** Ablation study results on the MR dataset.

MSFM	NSA	MR (Dice)	MR (Iou)
		0.8868	0.8396
✓		0.9196	0.8682
	✓	0.9027	0.8521
✓	✓	0.9386	0.8858

## Data Availability

To access the MR dataset, please contact Dr. Pang (pangshumao@126.com) and sign a confidentiality agreement Public CT dataset is available at https://github.com/MIRACLE-Center/CTSpine1K. The X-ray dataset is available at https://www.dropbox.com/scl/fi/80hduycgyrbse281sdtjf/scoliosis-xray-Single-View.zip?rlkey=opye7f5de0avh3isc1br5gwjh&e=2&dl=0, accessed on 1 Septermber 2024. The other data that support the findings of this study are available from the corresponding author, upon reasonable request.
